# Metataxonomic analysis and host proteome response in dairy cows with high and low somatic cell count: a quarter level investigation

**DOI:** 10.1186/s13567-023-01162-0

**Published:** 2023-04-04

**Authors:** Anja Ruud Winther, Vinícius da Silva Duarte, Davide Porcellato

**Affiliations:** Faculty of Chemistry, Biotechnology and Food Science, Norwegian University of Life Sciences, NMBU, Ås, Norway

**Keywords:** Shotgun proteomics, somatic cell, microbiota, weighted co-expression network analysis

## Abstract

Host response to invasive microbes in the bovine udder has an important role on the animal health and is essential to the dairy industry to ensure production of high-quality milk and reduce the mastitis incidence. To better understand the biology behind these host-microbiome interactions, we investigated the somatic cell proteomes at quarter level for four cows (collected before and after milking) using a shotgun proteomics approach. Simultaneously, we identified the quarter microbiota by amplicon sequencing to detect presence of mastitis pathogens or other commensal taxa. In total, 32 quarter milk samples were analyzed divided in two groups depending on the somatic cell count (SCC). The high SCC group (>100,000 cell/mL) included 10 samples and significant different proteome profiles were detected. Differential abundance analysis uncovers a specific expression pattern in high SCC samples revealing pathways involved in immune responses such as inflammation, activation of the complement system, migration of immune cells, and tight junctions. Interestingly, different proteome profiles were also identified in quarter samples containing one of the two mastitis pathogens, *Staphylococcus aureus* and *Streptococcus uberis*, indicating a different response of the host depending on the pathogen. Weighted correlation network analysis identified three modules of co-expressed proteins which were correlated with the SCC in the quarters. These modules contained proteins assigned to different aspects of the immune response, but also amino sugar and nucleotide sugar metabolism, and biosynthesis of amino acids. The results of this study provide deeper insights on how the proteome expression changes at quarter level in naturally infected cows and pinpoint potential interactions and important biological functions during host-microbe interaction.

**Supplementary Information:**

The online version contains supplementary material available at 10.1186/s13567-023-01162-0.

## Introduction

Whether or not the bovine intramammary environment contains a viable microbial community have been a topic of discussion for years [1, 2]. More evidence now suggest that opportunistic pathogens and commensal species are able to survive and thrive in a rather complex microbial environment in the udder [3–5]. There is also evidence that this community, together with the bacteria found on the teat skin, can protect the udder against invading bacteria that can cause mastitis. Certain species can protect the intramammary environment against pathogens by secreting antimicrobial substances. This includes coagulase negative staphylococci and *Corynebacterium* that are able to produce antimicrobials which prevent growth of known mastitis pathogens [6, 7]. In addition, lactic acid bacteria have been shown to adhere to mammary gland epithelial cells and modulate their production of pro-inflammatory cytokines [8]. Despite the protective role of the commensal microbiota of bovine milk, intramammary infections (IMIs) occur. IMIs caused by bacteria are the main cause of bovine mastitis. It results in redness, swelling, fever and discomfort for the cow, in addition to having a massive impact on the dairy industry. Milk from an infected cow has reduced quality and a changed composition of fat, protein, lactose and minerals, which affects milk properties and flavor [1]. Milk from a full blown mastitic infection is unfit for consumption, resulting in loss for both farmer and dairy industry. In addition to affecting the composition of the nutrients, a bacterial infection leads to an increase in the somatic cells in the milk. Both immune cells, such as macrophages and neutrophiles, migrate to the milk in order to fight of the infection, and the level of shedding of epithelial cells increase to rid the intramammary tissue of intracellular pathogens [1, 9]. This increase in the somatic cell count (SCC) in the milk is currently the main strategy to detect an infection. In a healthy udder, the SCC is typically between 10 000 and 100 000 somatic cells/mL. A case of mastitis is considered sub-clinical when the cow is without symptoms and the SCC is between 100,000 and 1 million cells/mL, and clinical in the event of the above-mentioned symptoms and a SCC of over 1 million cells/mL. These thresholds may differ between countries.

When a pathogenic bacterium enters the udder, the first line of defense it encounters is epithelial cells and macrophages. These recognize Pathogen Associated Molecular Patterns (PAMPs) through Toll-like receptor 2 and 4, and upon activation of said receptors, the NF-κB signaling pathway is initiated [10, 11]. The signaling pathway stimulates the production of pro-inflammatory cytokines and chemokines, resulting in the recruitment of additional immune cells to the udder lumen, such as neutrophiles [12, 13]. These are the first migrating immune cells to enter the udder lumen upon infection and engulf the bacteria they encounter. The efficiency of the activation of the NF-κB signaling pathway depends on the invading pathogen. Some strains of *Staphylococcus aureus* will induce mild and subclinical mastitis due to low production of cytokines IL-8 and IL-1β, while others will cause a higher production of these cytokines and hence clinical mastitis [14, 15]. Gram-negative pathogens such as *E. coli* stimulate a strong immune response in the udder and most cases of mastitis caused by this bacterium is the severe, clinical type [10]. In addition to the cytokines, the level of acute phase proteins such as cathelicidins and peptidoglycan recognition protein increase in the udder during the onset of an infection [16]. The same is true for members of the complement system, fibrinogen and antimicrobial mammary S100 calcium-binding proteins [17, 18].

There are several studies available that investigate the interaction between microbes, both commensals and pathogens, in bovine milk [5–8]. There are also various studies that address the interaction between pathogens and the immune cells of the udder [15, 19, 20]. This interaction decides the outcome of the infection: clearance or establishment of the pathogen. However, the interplay between the commensal microbial community and the somatic cells in the udder is not as extensively investigated. Knowledge regarding this is an important part of fighting mastitis as a healthy microbial community probably protects the udder against invaders. Lima et al. found that primiparous cows, as opposed to multiparous cows, harbors microbial diversity and taxonomic markers that likely contributes to a healthier udder [21]. Studies have also discovered that a greater richness of the udder microbiota makes the udder more resistant to infection [22–24]. These studies all put emphasis on the microbial composition of the udder. A different perspective would be to investigate the proteome of the somatic cells isolated from healthy udders and relate it to SCC and microbial composition. Typically, studies like this tend to focus on the whey proteins and caseins of different types of milk samples such as healthy, subclinical mastitis and clinical mastitis samples. The consensus seems to be that during an infection, proteins involved in immune-responses such as immunoglobulins, cathelicidins and members of the complement system increase in the milk [25–27], while the whey proteins and caseins decrease [28].

Less frequently found in the literature are studies concerning the proteins of the somatic cells in healthy udders and in cases of subclinical mastitis. Knowledge regarding the healthy udder, its cells and how they interact with the commensal microbiota will provide important information regarding avoidance of IMIs and possibly the early detection of an infection through biomarkers detectable in the milk. In this study the somatic cells of milk samples from healthy udders with high and low SCC (H-cows and L-cows, SCC above and below 100 000 cells/mL, respectively) were isolated and their proteome investigated. The objective of the study was to relate the proteome of the somatic cells to the level of SCC in the sample and the quarter microbial composition. Analysis uncovered clear differences between the proteomes of somatic cells from quarters with high and low SCC, with several proteins being differentially expressed between the two conditions. Weighted correlation network analysis (WGCNA) revealed three groups of co-expressed proteins with a positive correlation to SCC which included different aspects of the immune response, but also amino sugar and nucleotide sugar metabolism, and biosynthesis of amino acids.

## Materials and methods

### Cows and sample collection

Four Norwegian Red cows were selected from “The Livestock Production Research Centre” at the Norwegian University of Life Sciences based on a previous study by Winther et al. [4]. The study categorized the cows as H- or L-cows based on high (>100 000 SCC/mL) or low (<100 000 SCC/mL) somatic cell count, respectively, at the start of the study as recorded by the automatic milking system (Delaval Online Cellcounter). For this project, two H-cows (H3 and H5) and two L-cows (L2 and L4) were chosen for further sampling. The cattle enrolled in the study were housed in freestall cubicles with bedding materials of rubber mats with raw wood chips. Their diet consisted of silage and pelleted feed. Two samples were collected from each quarter, one before and one after the regular milking routine (32 independent quarter samples), on one occasion (April 2021). To reduce the risk of including environmental bacteria in the samples, the teats were washed with iodine and then alcohol, and 200 mL milk was collected manually. The “Procedure for Collecting Milk Samples” of the National Mastitis Council (NMC) was followed. The milk samples were stored on ice until their arrival in the laboratory (within 2 h after the last sample was collected) where they were processed for analysis immediately. No invasive procedures were used in this study. Permission for both sample collection and use of information regarding the samples were given by the farm owners. The farm operates under the regulations of the Norwegian Food Safety Authority regarding food production and animal care. The Norwegian Cattle Health Recording System provided additional metadata for each of the cows [29].

At arrival in the laboratory, 0.1 mL raw milk from each sample where plated on TSA blood agar plates (ThermoFischer Scientific, Massachusetts, USA). The plates were then incubated at 37 °C under aerobic conditions for 24 h. After plate counting, the quarters with > 10 colonies per 0.1 mL (mixed or pure) were labeled as having an IMI. This was based on definition “A” of Dohoo et al. [29]. Milk from all samples were also sent to the Tine Laboratory in Heimdal and analyzed with Bentley FTS (Bentley Instrument Inc, Chaska, MN, USA) for somatic cells, fat, protein, lactose, urea, and FFA content.

### DNA extraction, amplicon sequencing and sequence analysis

For analysis of microbiota in the milk samples, bacterial pellet was obtained and amplification and sequencing of the 16 S rRNA genes were performed as described previously [3]. The raw sequencing data were analyzed with the Dada2 algorithm as described by Winther et al. [4].

### Suspension trapping and liquid chromatography with tandem mass spectrometry (LC-MS/MS)

To prepare peptides for identification through LC-MS/MS, 40 mL milk was processed the same way as for DNA extraction for amplicon sequencing with an additional washing step with 2% citrate water in order to rid the samples of as much of the milk caseins as possible. The final pellet was resuspended in 200 µL lysis buffer (50 mM TrisHCl, pH 7.5, 4% SDS, 10 mM DTT) and the cells were lysed by bead beating (0.2 g of 106 μm acid washed beads, Sigma). The resulting protein solution was denatured at 95 °C for 10 min and alkylated with 100 mM iodoacetamide for 20 min in the dark. Phosphoric acid (1/10^th^ of the total volume) was used to acidify the sample before loading it onto the suspension trapping column for washing and digestion as previously described [30]. Briefly, the columns, made up of a plug of C18 (Empore) with an overlaying stack of quartz (Munktell MK360 quartz filter), were made in a 200 µL pipette tip (Eppendorf™ epT.I.P.S.™, 2-200 µL). The protein solution was loaded onto the column in a suspension trapping buffer containing 90% methanol and 100 mM Tris-Cl pH 7.1, resulting in a protein suspension on top of the quartz stack. Centrifugation trapped the protein suspension in the quarts stack where it is washed twice (suspension trapping solution and 50 mM ammonium bicarbonate, respectively) and digested with Trypsin (1:00 enzyme/protein ratio) (V5111, Promega) in 50 mM ammonium bicarbonate for an hour at 47 °C. Additional washes with 0.5% and 0.1% trifluoroacetic acid (TFA) moved the peptides to the C18 plug, where an elution buffer (80% acetonitrile, 0.1% TFA) eluted the peptides from the column. Analysis of the peptides were performed as previously described by Myrbråten et al. with a nano UPLC (nanoElute, Bruker) coupled to a trapped ion mobility spectrometry/quadrupole time of flight mass spectrometer (timsTOF Pro, Bruker) [31]. The proteomics data have been deposited to the Proteomics Identification Database (PRIDE) with accession number PRIDE: PXD035328.

### Label free quantification analysis of mass spectrometry data

The raw ms/ms data were analyzed using the MaxQuant software [32] with the MaxLFQ algorithm [33] for label free quantification. The search database was taken from UniProt Proteomes (*Bos taurus*, UP000009136). The “match between runs” option of MaxQuant was used to increase the number of hits. All identifications were filtered to achieve a protein FDR score of 0.01 and a min. peptide count of 1. For further analysis we used the proteinGroups output file from MaxQuant. The filtering steps were performed with the Perseus software [34], removing hits considered as contaminants (Reverse, Only identified by site, and Potential contaminants). The caseins detected in the samples were removed with the contaminants. Finally, for differential expression and WGCNA analysis, a protein was considered to be present in a sample only if it was detected in both replicates.

### Differential expression analysis

Differentially expressed proteins were identified using the DEP package in R [35]. Missing values were imputed using random draws from a Gaussian distribution centered around a minimal value (NMAR) utilizing q = 0.01. Significant proteins were detected using 0.05 as the adjusted *p*-value and 1.5 as the threshold for the log_2_ fold change.

### Weighted correlation network analysis (WGCNA)

In order to detect modules of proteins with the same expression patterns between high and low SCC, the WGCNA package was utilized as described by Zhang et al. with a soft threshold power of β = 9 [36]. Modules were identified by using a dynamic tree-cutting algorithm with parameters: minimal module size = 20, deepSplit = 2, and merge cut height = 0.2. Module membership (kME) was determined by calculating Pearson correlation between each protein and each module eigenprotein and the corresponding *P*-values [37].

### KEGG enrichment pathways and gene ontology enrichment analysis

KEGG enrichment pathway analysis of the differentially expressed proteins were performed with the clusterProfiler R package. Only significant proteins were used in the analysis (*p* = 0.05, foldchange = 1.5). Gene ontology enrichment analysis was performed with the UniprotR package.

## Results

### Amplicon sequencing

**Figure 1:**
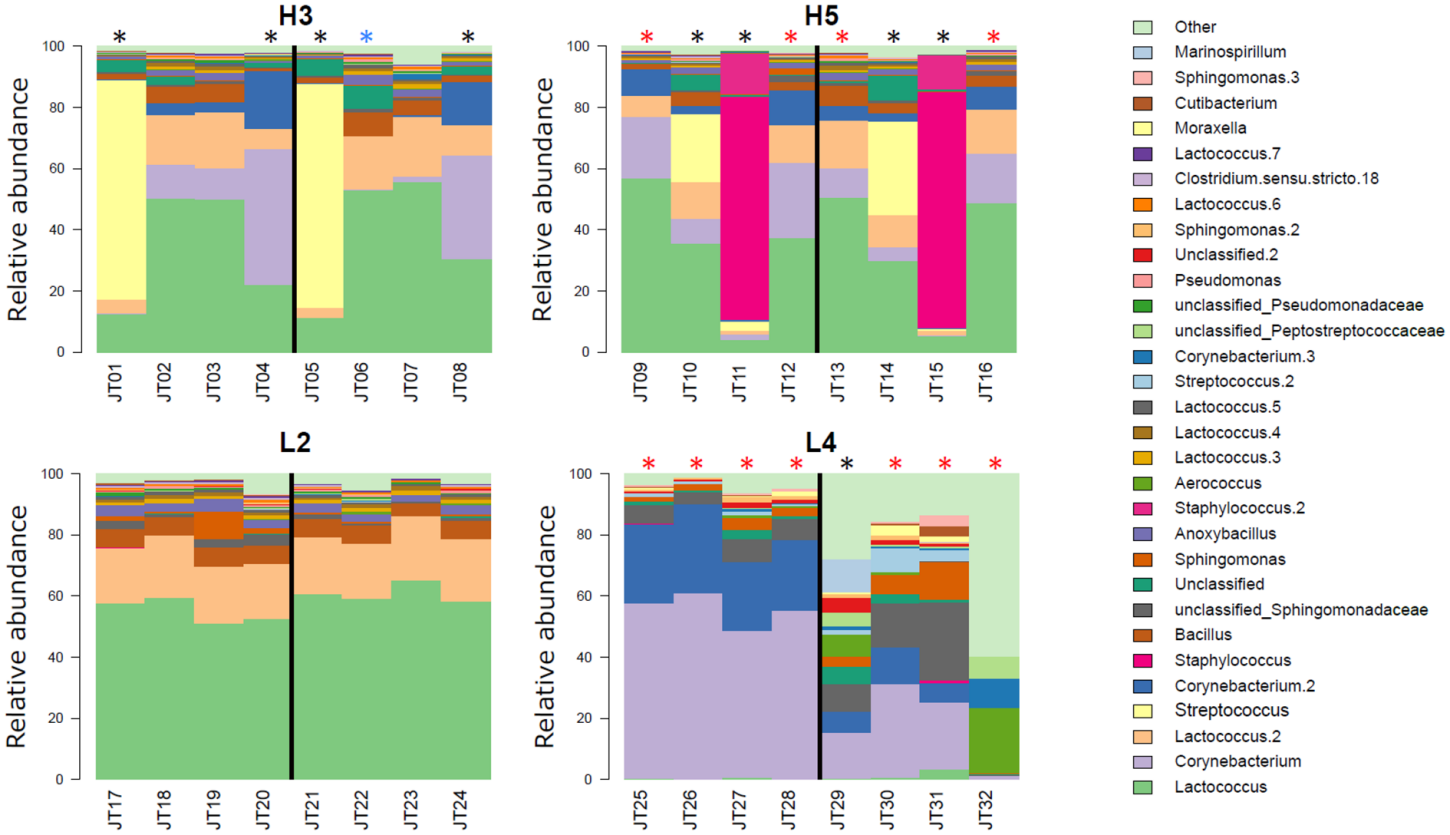
**
Microbial composition of the 32 milk samples based on 16 S amplicon sequencing**. Cow H3 and H5 refer to the two cows with a high SCC from the previous study by Winther et al., while cow L2 and L4 refer to the two cows with a low SCC from the same study. Asterisk above bars indicate a recorded IMI (red), SCC > 100,000 cells/mL during this study (blue), or both (black). Labelling a sample with an IMI is based on cultivation on blood agar where samples with > 10 colonies per 0.1 mL is considered to harbor an IMI (definition “A” by Dohoo et al. [38]).

The four cows included in this project were defined as H-cows (SCC > 100,000 cells/mL) or L-cows (SCC < 100,000 cells/mL) based on a previous study conducted by Winther et al. where the SCC were measured over three consecutive days at the start of the project [4]. The study revealed a difference in the stability and composition of the udder microbiota between H- and L-cows, where the H-cows displayed an imbalanced microbiota and a lower diversity over time compared to the L-cows. The imbalance was caused by *Staphylococcus* or *Corynebacterium*. With the purpose of studying the proteomic profile of the somatic cells from samples with high and low SCC, we chose two cows defined as H-cows (H3, H5) and two cows defined as L-cows (L2, L4) from the previous study and collected quarter milk samples from these. The milk was collected before and after regular milking at quarter level in an attempt to acquire milk from the teat cistern and from deeper parts of the udder, respectively. The incentive was that the milk collected after regular milking would contain less environmental contaminants as these would be flushed away during the milking procedure. Initial investigations of the composition of the udder microbiota with amplicon sequencing of the 16 S rRNA genes (Figure 1) showed that this was the case for *Corynebacterium* in cow L4. Samples JT25-JT28, taken before the regular milking, contained up to ~90% *Corynebacterium*, most of which was not present in the samples taken after regular milking (samples JT29-JT32).

The quarter samples with a SCC above 100,000 cells/mL were regarded as having a high SCC (labeled with blue or black asterisk in Figure 1). Those samples made up 10 of the 32 samples collected, and 90% of these were from the H-cows. Samples were labeled with an IMI if upon cultivation of 0.1 mL on blood agar > 10 colonies appeared on the plate. This definition of an IMI is by Dohoo et al. [38]. 20 samples were labeled with an IMI (red or black asterisk in Figure 1), 60% of these samples were from the H-cows. Both H-cows had an imbalanced microbial community in one quarter, caused by *Streptococcus uberis* (H3) or *Staphylococcus aureus* (H5). One sample, JT24, contained blood. As no imbalance in the microbiota was detected in this sample, which is usually the case during a bacterial infection [3, 4], the blood could be a result of a yeast infection or trauma to the udder [39].

### General proteomics analysis revealed a clustering of samples with a high SCC

**Figure 2:**
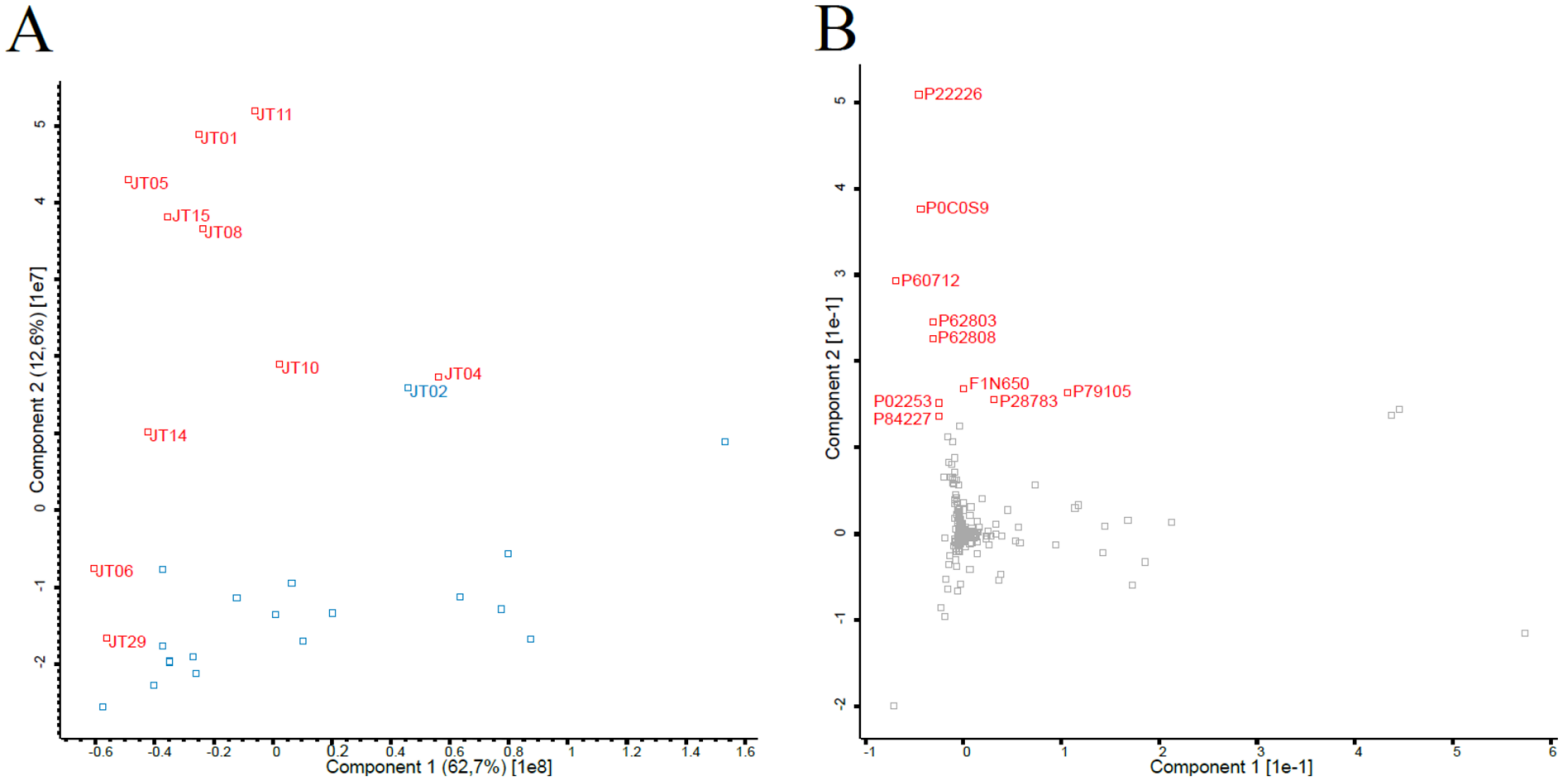
**General analysis of the proteomics data**. **A** PCA plot of the whole proteome dataset of the 32 milk samples. Red datapoints represent samples with high SCC (>100 000 cells/mL), blue datapoints represent samples with low SCC (<100 000 cells/mL). Sample JT24 was omitted from the plot. Sample JT02 cluster with the high SCC samples despite being considered a low SCC sample. **B** Overview of the proteins that were responsible for the variation in the second component of the PCA. P0C0S9, P62803, P62808, P02253, and P84227 are histones, P60712 and F1N650 are actin, and P22226, P28783, and P79105 are proteins related to an immune response.

With the intent to investigate how the cows in the study respond to the bacteria present in their udders, somatic cells were isolated from the quarter milk samples and studied with liquid chromatography coupled with tandem mass spectrometry (LC-MS/MS). The raw data were analyzed with the MaxLFQ algorithm in MaxQuant [32, 33] and filtered with Perseus [34]. A total of 5427 proteins were detected in the samples. The PCA pot in Figure. 2A show that the samples with a SCC > 100 000 cells/mL clustered in the upper and left part of the plot (labeled in red). In the PCA plot, sample JT24 was excluded. As the sample contained blood, the proteomic dataset was heavily influenced by hemoglobin, leading the other samples to cluster tightly together and no apparent pattern to appear. Sample JT02 was similar to the samples with high SCC in the PCA plot. This sample had a SCC below, but close to, 100 000 cells/mL which was chosen as threshold to differentiate the two groups. The variation in the second component of the PCA plot was mainly driven by histones (P0C0S9, P62803, P62808, P02253, P84227) and actin proteins (P60712, F1N650) present mostly in the high SCC samples. In addition, proteins P22226, P28783, and P79105 were also correlated with high SCC samples and are proteins involved in immune responses and regulation of inflammatory processes (Figure 2B).

### Differential expression analysis uncovers a specific expression pattern in high SCC samples

**Figure 3:**
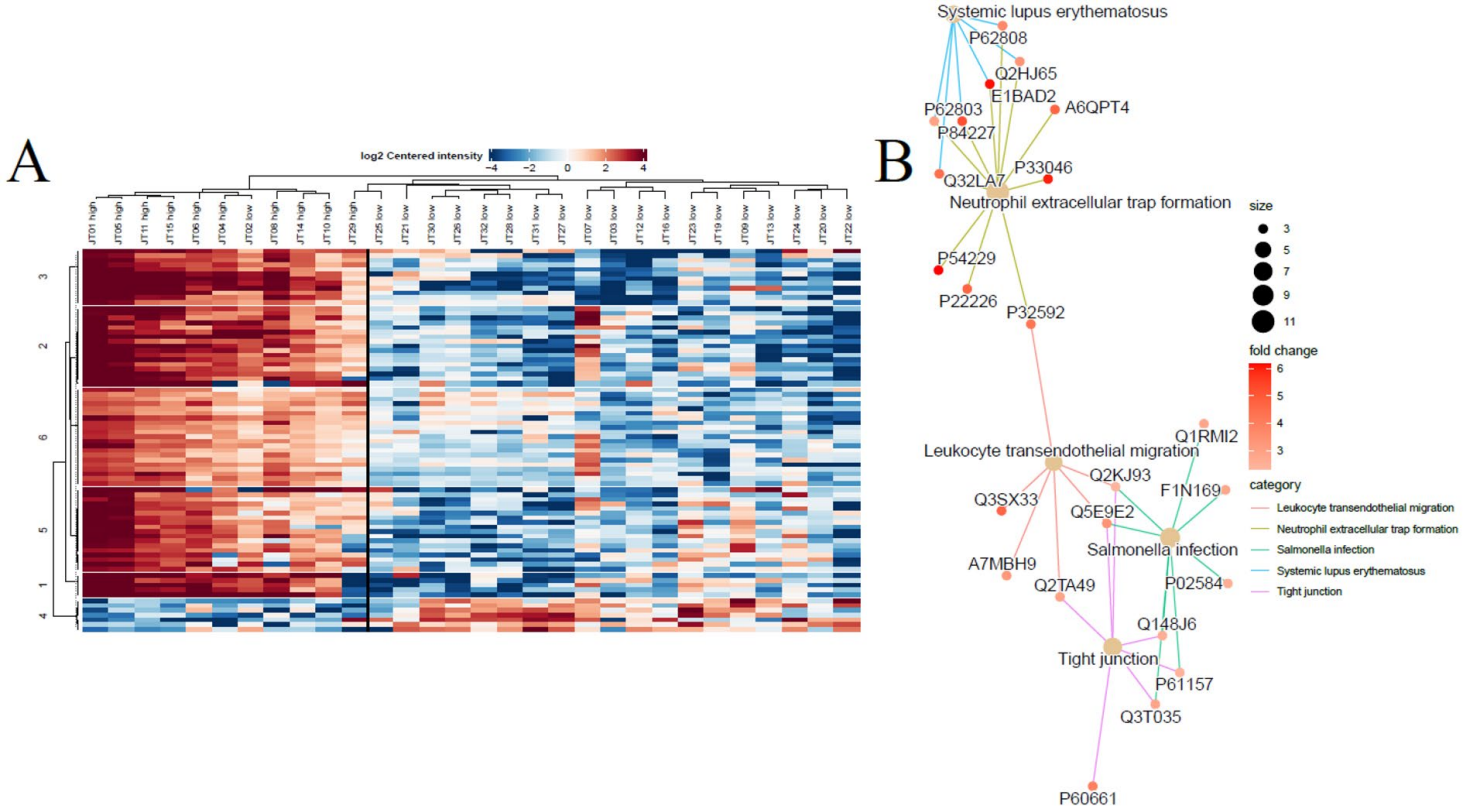
**
Differential expression analysis of the quantitative proteomics dataset**. **A** The samples with a high SCC (>100,000 cells/mL, labeled as such) cluster to the left side of the heatmap. The black line separates the high and low SCC samples. The 80 proteins making up the rows are divided into 6 k-means consisting of proteins with a similar expression pattern. **B** KEGG enrichment analysis of the 73 differentially expressed proteins enriched in the high SCC samples.

Analysis of differential expression between high and low SCC samples was carried out by considering the same quarter before and after milking as replicates. A protein was only considered to be present in a sample if it was detected in both replicates. The filtered protein list contained 2372 proteins. Differential expression analysis of the quantitative proteomics data utilizing a threshold of +/− 1.5 fold change in high SCC over low SCC (*p* < 0.05) revealed 80 proteins to be differentially expressed between the two conditions. The heatmap in Figure 3A displays the differentially expressed proteins grouped into 6 k-means that separates proteins based on general expression pattern. The samples with a high SCC are clustered together on the left side of the heatmap, separated from the low SCC samples with a black line. Sample JT02 clustered with the high SCC samples for the same reason as mentioned above – a SCC close to the 100,000 cells/mL threshold set to separate samples of high and low SCC. It is noteworthy to mention that although samples JT29 and JT25 have clustered with the samples with high and low SCC, respectively, “JT29 high” belongs to the same arm as samples classified as low SCC following hierarchical clustering. We believe that the overall low expression level observed specifically for this sample can be due to a different somatic cell profile before and after milking.

Of the 80 proteins that were differentially expressed between the two conditions, 73 were enriched in the high SCC samples, while 7 were downregulated in the same samples and enriched in the low SCC samples. Further investigations of the 73 proteins enriched in the high SCC samples revealed that they were involved in biological processes such as “innate immune response”, “phagocytosis”, “defense response to Gram-positive bacterium”, “cell migration”, and “antimicrobial humoral immune response mediated by antimicrobial peptide”. KEGG pathway enrichment analysis of the same 73 proteins (Figure 3B) revealed pathways involved in immune responses such as inflammation, activation of the complement system, migration of immune cells, and tight junctions. These data agree with the proteins responsible for the clustering of the high SCC samples in the PCA plot in Figure 2A and B.

**Figure 4:**
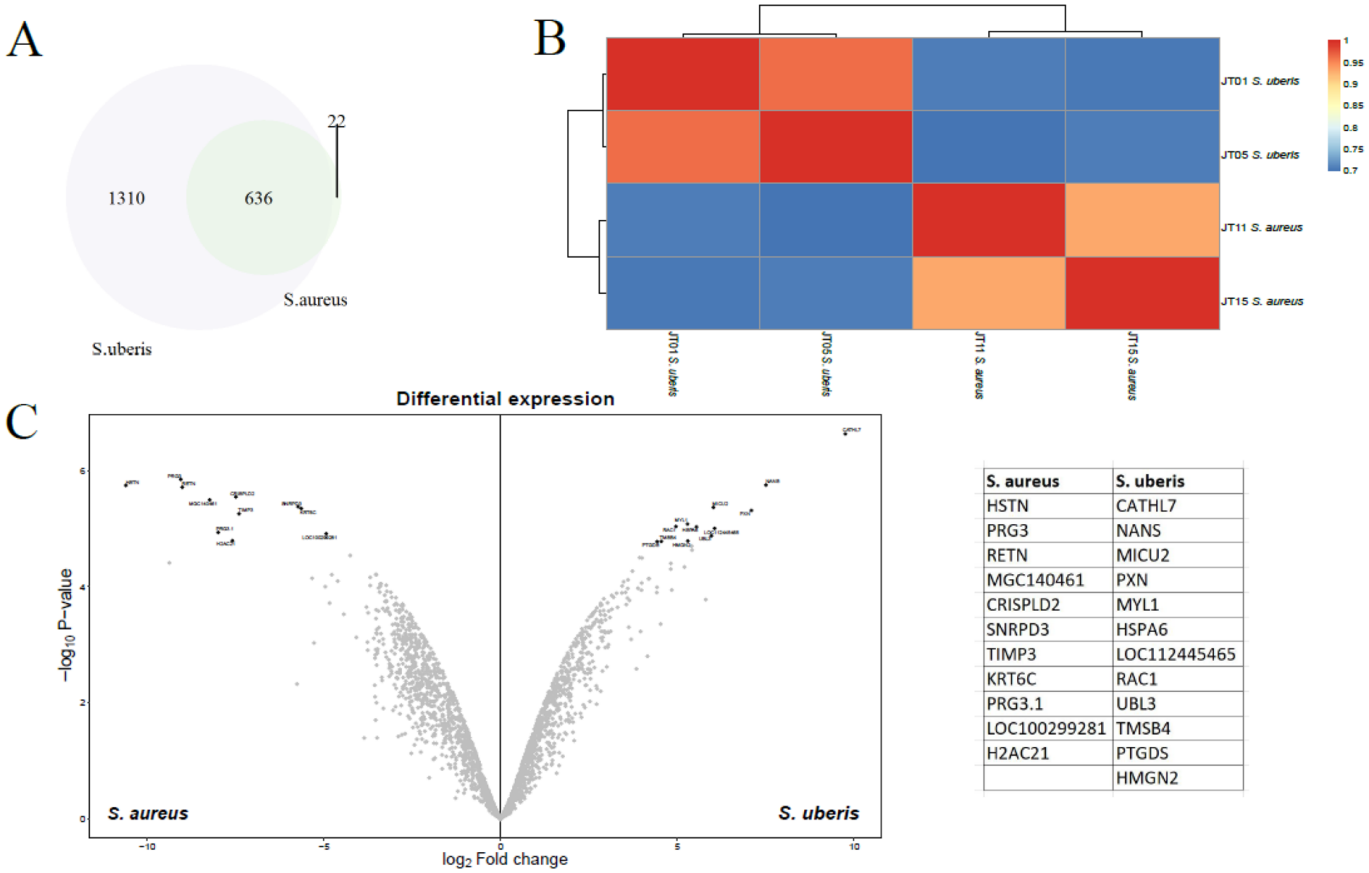
**Investigation of the proteomes of the quarters infected with *S. aureus*** **and *S. uberis***. **A** Venn diagram showing the overlap between the proteomes. 636 proteins were detected in both proteomes, 1310 proteins were only found in the quarter infected with *S. uberis*, and 22 proteins were detected only in the quarter infected with *S. aureus*. **B** Heatmap depicting the correlation between the 4 samples. **C** Differential expression analysis revealed 11 proteins enriched in the quarter infected with *S. aureus* and 12 proteins enriched in the quarter infected with *S. uberis*.

Figure 4 A shows the overlap between the proteomes of the quarter infected with *S. aureus* and the quarter infected with *S. uberis*. The quarters had 636 proteins in common. The proteome of the quarter infected with *S. uberis* was considerably larger (1946 proteins) than the proteome of the quarter infected with *S. aureus* (658 proteins). The heatmap depicting the correlation between the samples (Figure 4B) shows a low correlation between the proteomes of the infected quarters. Differential expression analysis with the same parameters as utilized above revealed a list of 11 proteins enriched in the quarter infected with *S. aureus* and 12 proteins enriched in the quarter infected with *S. uberis* (Figure 4C; Table 1).

**Table 1:** **
Proteins that were found to be enriched in quarter milk samples  infected with** ***Staphylococcus aureus*** **and** ***Streptococcus uberis. ***

Protein ID	Protein name	Biological process
***Staphylococcus aureus***		
HSTN	Histatherin	Defense response to bacterium
PRG3	PRG3 protein	Immune response
RETN	Resistin transcript variant 1	Aging/hormone response
CRISPLD2	Cysteine-rich secretory protein LCCL domain-containing 2	Extracellular matrix organization
MGC140461	MGC140461 protein	Immune response
TIMP3	Metalloproteinase inhibitor 3 - negative regulation of endopeptidase activity/response to cytokine/hormone.	
PRG3.1	PRG3 protein	Immune response
H2AC21	Histone H2A	
SNRPD3	Small nuclear ribonucleoprotein Sm D3 - spliceosomal snRNP assembly	
KRT6C	IF rod domain-containing protein	Keratin
LOC100299281	PKS_ER domain-containing protein - oxidoreductase activity?	
***Streptococcus uberis***		
CATHL7	Cathelicidin-7	Antimicrobial humoral immune response mediated by antimicrobial peptide
NANS	N-acetylneuraminate synthase	Carbohydrate biosynthetic process
MICU2	Calcium uptake protein 1, mitochondrial	Calcium import mitochondrium
PXN	Paxillin - cell-matrix adhesion/endothelial cell migration	
LOC112445465	Histone H2B	Nucleosome assembly
MYL1	Myosin light chain 1/3, skeletal muscle isoform	Muscle contraction
HSPA6	Heat shock protein family A (Hsp70) member 6	Cellular response to heat
UBL3	Ubiquitin-like protein 3	
RAC1	Ras-related C3 botulinum toxin substrate 1	Actin filament organization / cell motility
TMSB4	Thymosin beta-4	Actin filament organization
PTGDS	Prostaglandin-H2 D-isomerase	Mast cell degranulation
HMGN2	Non-histone chromosomal protein HMG-17	Chromatin organization

### Weighted correlation network analysis uncovers 12 modules of similarly expressed proteins

**Figure 5:**
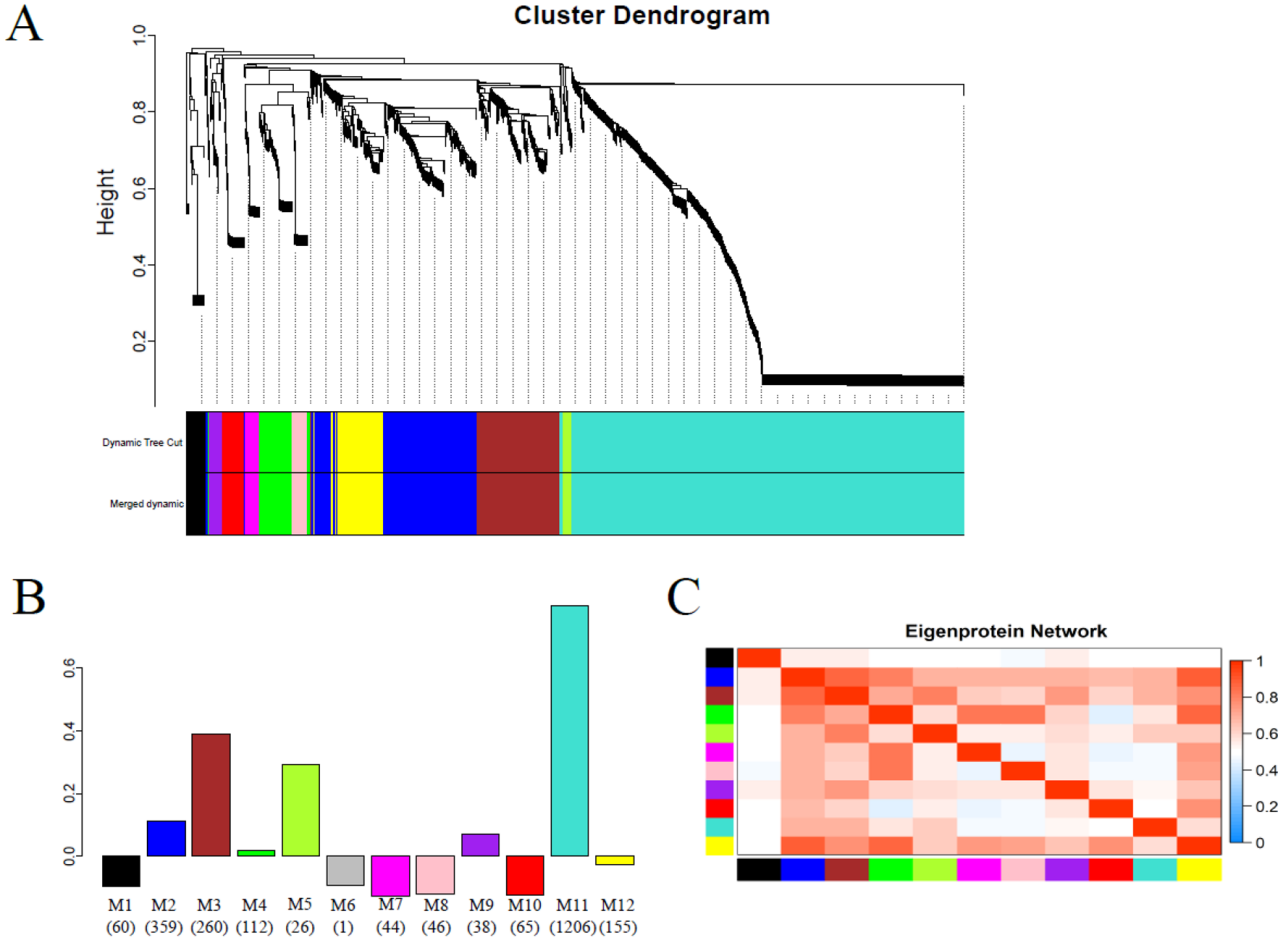
**WGCNA analysis of the quantitative proteomics dataset**. **A** Cluster dendrogram generated by unsupervised hierarchical clustering of the proteomic dataset based on topological overlap followed by branch cutting uncovered 12 modules indicated by different colors. **B** Bar chart displaying the correlation between the modules (M1-M12) and SCC. The number of proteins included in a module is indicated in parenthesis under each bar. **C** Heatmap displaying the correlation between the modules.

In order to explore co-expression networks in our proteome dataset, we utilized weighted correlation network analysis (WGCNA) on the dataset of 2372 proteins filtered based on replicates. This type of network analysis uses pairwise correlation relationships of proteins and their topological overlap to organize the proteome into a network of biologically meaningful modules of co-expressed proteins [40–42]. The WGCNA analysis of the proteomics dataset resulted in twelve modules, M1-M12, where the smallest contained one protein (M6) and the largest contained 1206 proteins (M11) (Figures 5A and B). Correlation analysis of the module eigenprotein value with SCC showed 3 modules to be highly correlated (≥ 0.3). Module M11, which was the largest module and contained 1206 proteins had the highest correlation with the SCC (0.80), while module M3 and M5 had a correlation score of 0.39 and 0.30, respectively. The correlation between modules is displayed in Figure 5C. Proteins described by PCA and differential abundance to be enriched in the high SCC samples were also found in these 3 modules. The top 10 GO terms of modules M3, M5 and M11 are displayed in Additional file 1, while the hub proteins (proteins that best represent the module) are reported in Additional file 2.

## Discussion

Mastitis is the result of host response to eradicate invading microorganisms specially members of the domain Bacteria [43]. From the host perspective, the innate immune system is considered the first line of defense against bacteria and is mediated by macrophages, neutrophils, natural killer cells (NK) and cytokines [44]. These components play a crucial role in the inflammatory response in the udder, with polymorphonuclear neutrophils (PMNs) representing one of the most abundant leukocyte cells [45]. In a mastitic mammary gland, PMNs constitute up to 90% of the total milk leukocytes, whereas in healthy mammary gland macrophages are the predominant cell type and account for 35–79% of total leukocytes in milk [46].

Overall, few studies up to date have explored changes in the expression and production of proteins from somatic cells during an ongoing IMI, which ultimately can be used as potential biomarkers in the diagnosis of mastitis [15, 47]. The robustness of the immune response is highly dependent of the mastitis-causing agent and major pathogens such as streptococci, *S. aureus*, and coliforms can lead to a significant increase in milk SCC, whereas minor pathogens (Coryneforms and coagulase-negative staphylococci) are associated with lower SCC [48]. In fact, metataxonomic analysis of milk samples with low SCC (Animal L4, samples JT25-JT32) evidence a high prevalence of *Corynebacterium* across all quarters, primarily before milking (samples JT25-JT28).

In the current study we have compared the proteomes of somatic cells isolated from bovine quarter milk samples with high and low SCC. PCA analysis showed clustering of the high SCC samples driven by histones, actin, and 3 immune-related proteins previously shown to be upregulated in high SCC samples. The first of these three, cathelicidin (P22226), is part of the first line host defense and its upregulation is typically detected during an *S. uberis* or *S. aureus* infection [16, 27], and generally in samples with a high SCC [26]. In our study, we found cathelicidin to be not only responsible for the clustering of the high SCC samples in the PCA plot and enriched in these samples when analyzing differential expression between the two conditions, but also to be enriched in the quarter samples infected with *S. uberis*.

The other two immune-related proteins responsible for the clustering in the PCA plot were Protein S100-A9 (P28783) and Protein S100-A12 (P79105). These are both calcium- and zinc-binding proteins involved in the inflammatory process. Overall, adequate minerals levels are required for the maintenance of a healthy udder and their deficiency can increase the risk of mastitis mainly due to the reduced activity of immune cells or malfunction of teat innate defense mechanisms [49]. The protein 100-A9 is usually part of a larger complex called calprotectin, a component in Neutrophil Extracellular Traps (NETs) [50]. These traps are created by degranulated neutrophiles, a process where neutrophiles release extracellular fibers, creating a meshwork that trap and kill both Gram-positive and Gram-negative bacteria [51]. The establishment of NETs can also occur outside the mammary gland, such as in the blood. By measuring neutrophil extracellular trap (NET)-related variables in the serum of Holstein dairy cows during the transition period, Jiang et al. suggested a positive association between blood NET formation, high somatic cell count, and postpartum mastitis risk [52]. Neutrophil transcriptome between high- and low-SCC cows revealed differences in genes involved in both the cell cycle and NETosis.

In addition to seeing this protein as important for the clustering in the PCA plot, NETs appeared as an KEGG enrich pathway when analyzing proteins enriched in the high SCC samples. Another important component of NETs are nucleosomes which are made up of DNA and histones. Brinkmann et al. found that they were able to stain NETs with antibodies against histones H1, H2A, H2B, H3, and H4 [51]. The histones we found as being important for the clustering of our PCA plot were H2A, H4, H2B, H1.2, H3.2. Several of these have also been found to be upregulated in high SCC samples previously [16]. Protein S100 A12 has also been found in relation to heightened SCC in previous studies [16, 53], but apart from being part of the immune response, its direct function seems to be unknown. According to Wei et al. NETs and their component histone showed a significant cytotoxic effect to bovine mammary epithelial cells in vitro [54]. The authors suggested that histone has a substantial role in BMEC damage, as well as could be involved in the induction of necrosis and apoptosis of BMECs through the activation of caspase 1, caspase 3, and NLRP3 [54]. In this study, by adopting WGCNA analysis, several proteins classified in the GO term “apoptotic process” was found in the largest module of co-expressed proteins (module M11), which displayed the highest correlation with the SCC.

The positive regulation of the extracellular signal-regulated kinase 1/2 (ERK1/2) cascade (module M5) and the negative regulation of cell population proliferation (module M11) are cellular processes commonly reported during IMI and mastitis development. The ERK1/2 cascade is a central signaling pathway that regulates a wide variety of stimulated cellular processes, such as proliferation, differentiation, survival, apoptosis, and stress response [55]. Several studies have showed that the modulation of ERK and MAPK signaling pathway can lead to anti-inflammatory role in mastitis due to the decreased release of pro-inflammatory cytokines such as tumor necrosis factor-α, IL-1β, and IL-6 [56–59].

The enrichment of proteins classified in the biological processes “positive regulation of lamellipodium assembly” and “Arp2/3 complex-mediated actin nucleation” (both grouped in module 5) in high-SCC samples indicate lamellipodia extension and filopodia formation during cell migration, which is important to new adhesion contacts, motility and spreading [19, 20, 60, 61]. By evaluating the proteomic changes in the mammary tissue of rats challenged with *S. aureus*, Cai et al. observed that the upregulation of both pathways is a host-response to fight against bacteria to reduce cellular death and tissue injury caused by IMI [62].

Comparative analysis of differentially abundant proteins found between quarter milk samples containing *S. aureus* or *S. uberis* reveals that three immune-related proteins might be involved in specific host response according to the mastitis causing-pathogen. Histatherin (HSTN) and proteoglycan 3-like (PRG3) were identified as biomarkers of a quarter sample infected by *S. aureus*, whereas cathelicidin-7 (CATHL7) was overrepresented in a quarter infected with *S. uberis*. Histatherin is a 6 kDa antimicrobial protein (a chimera of histatin and statherin) naturally present in cow’s milk and associated with mastitis prevention and reduced infection in the newborn calf [63, 64]. With regards to PRG3, this proteoglycan has been implicated in granulocyte activation and histamine biosynthetic process, playing a wide and fundamental role in inflammatory response in different pathological processes [65, 66]. In a quarter infected by *S. uberis*, the identification of the protein CATHL7 stands out. This molecule belongs to a heterogeneous class of host defense peptides (HDP) with antimicrobial activity and potent chemotactic function released in milk [67]. Higher levels of cathelicidin have also been identified in milk samples obtained of quarters infected by *Streptococcus agalactiae*, but not when *Serratia* spp. was involved, which indicates the different ability of microorganisms to induce cathelicidin release in milk [68].

The use of label-free shotgun proteomics proved to be a useful and promising tool in the identification and differentiation of putative biomarkers for the diagnosis of bovine mastitis [69], a multifactorial disease in which the host’s immune response occurs at the quarter level and is dependent of the bacterial species involved. As with the majority of studies, the design of the current study is subject to limitations and results must be interpreted with caution due to the reduced number of milk samples with high SCC and positive for *S. aureus* and *S. uberis*. In addition, shotgun proteomics is a bottom-up approach which rely on the quality of the database used to identify the proteins from peptides. Here, we used the reference genome of *Bos Taurus*, originated from a Hereford breed. The milk in this study was collected from the Norwegian Red breed which might contain different proteins not identified. Lastly, this study considered all sub-populations of somatic cells and their protein profile regardless the infection stage (i.e., recent IMI or resolution phase).

Our data demonstrate a clear separation in protein expression between cows with high and low SCC, and we identified specific protein profiles in the two groups. These were in good correlation with microbiota analysis and presence of mastitis pathogens. We showed the existence of a change in expression depending on the infecting pathogens, and that during a heightened SCC the co-expression of proteins belonging to three modules changed. These modules were positively correlated with high SCC. By studying quarter milk samples obtained directly from cows regularly milked for production, we were able to describe the biological system in natural infected cows and not in model system (such as cell lines or peripheral neutrophil cells). This study might open new prospective for the characterization of the SCC subpopulation in order to attribute the expressed protein to the type of cell.

## Supplementary Information

**Additional file 1. The top 10 GO terms for the proteins of modules M3, M5 and M11**. Weighted correlation network analysis of the 2372 proteins resulted in 12 modules of co-expressed proteins. The figure displays the top 10 GO terms for the proteins of the three modules that were highly correlatedto the somatic cell count (M3, M5, M11).

**Additional file  2.** **The hub proteins of modules M3, M5 and M11**. Hub proteins for the three modules with the highestcorrelation with the somatic cell count and their respective GO biologicalprocess. The hub proteins of a module indicate the proteins that best represents that specific module. 

## Data Availability

The fastq files have been deposited at the European Nucleotide Archive with accession number PRJEB54099.
