# Supplementary figures and images for: Metataxonomic analysis and host proteome response in dairy cows with high and low somatic cell count: a quarter level investigation
Source: Vet Res. 2023 Apr 4;54:32. doi: 10.1186/s13567-023-01162-0 (PMC10074679; doi:10.1186/s13567-023-01162-0)

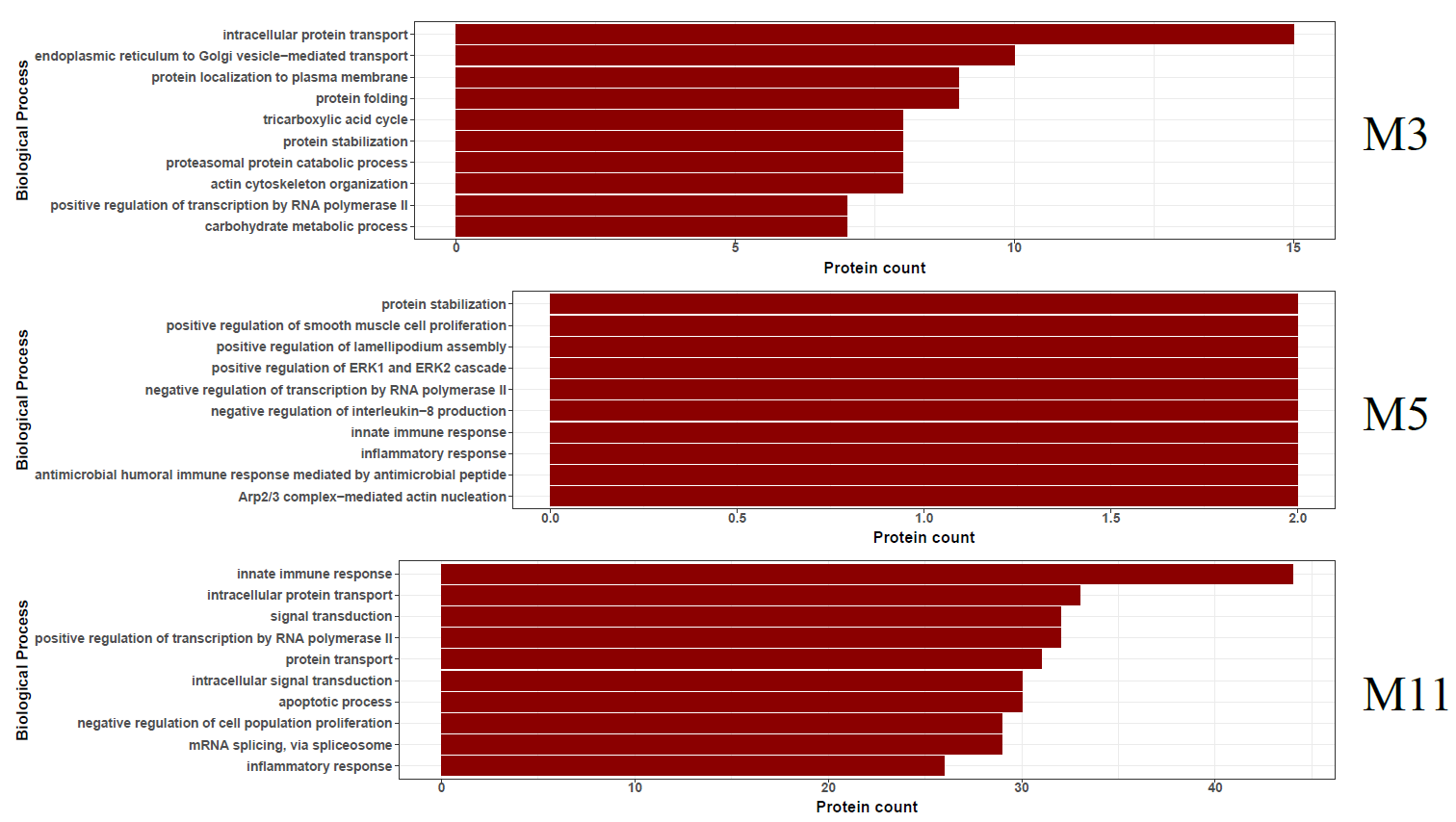

Supplement: Supplementary file 1 — Additional file 1. The top 10 GO terms for the proteins of modules M3, M5 and M11. Weighted correlation network analysis of the 2372 proteins resulted in 12 modules of co-expressed proteins. The figure displays the top 10 GO terms for the proteins of the three modules that were highly correlatedto the somatic cell count (M3, M5, M11). [file 13567_2023_1162_MOESM1_ESM.docx]
