# Supplementary material for: Metataxonomic analysis and host proteome response in dairy cows with high and low somatic cell count: a quarter level investigation
Source: Vet Res. 2023 Apr 4;54:32. doi: 10.1186/s13567-023-01162-0 (PMC10074679; doi:10.1186/s13567-023-01162-0)
Supplement: Supplementary file 2 — Additional file 2. The hub proteins of modules M3, M5 and M11. Hub proteins for the three modules with the highestcorrelation with the somatic cell count and their respective GO biologicalprocess. The hub proteins of a module indicate the proteins that best represents that specific module. [file 13567_2023_1162_MOESM2_ESM.docx]

| **M3** | | **M5** | | **M11** | | |
| --- | --- | --- | --- | --- | --- | --- |
| **Protein** | **Biological process** | **Protein** | **Biological process** | **Protein** | **Biological process** |  |
| Dynamin GTPase | negative regulation of membrane tubulation | Vimentin | astrocyte development | Phospholipid-transporting ATPase | aminophospholipid transport |  |
| Citrate synthase, mitochondrial | carbohydrate metabolic process | Annexin | actin cytoskeleton reorganization / adaptive immune response | Docking protein 3 | Ras protein signal transduction |  |
| Hypoxia up-regulated 1 | cellular response to hypoxia | Cathelicidin-1 | antimicrobial humoral immune response mediated by antimicrobial peptide | Sec1 family domain containing 1 | endoplasmic reticulum to Golgi vesicle-mediated transport |  |
| NAD(P) transhydrogenase, mitochondrial | NADPH regeneration | Alpha-actinin-4 | peroxisome proliferator activated receptor signaling pathway | Prefoldin subunit 2 | negative regulation of amyloid fibril formation |  |
| Lamin B2 | structural constituent of cytoskeleton | Adenylyl cyclase-associated protein | actin cytoskeleton organization | Leucine zipper and CTNNBIP1 domain-containing protein | ? |  |
